# An Interactive Review on the Role of Tocotrienols in the Neurodegenerative Disorders

**DOI:** 10.3389/fnut.2021.754086

**Published:** 2021-10-26

**Authors:** Ruth Naomi, Nurul Husna Shafie, Priyatharisni Kaniappan, Hasnah Bahari

**Affiliations:** ^1^Department of Human Anatomy, Universiti Putra Malaysia, Serdang, Malaysia; ^2^Department of Nutrition, Faculty of Medicine & Health Sciences, Universiti Putra Malaysia, Serdang, Malaysia; ^3^UPM-MAKNA Cancer Research Laboratory, Institute of Bioscience, Universiti Putra Malaysia, Serdang, Malaysia; ^4^Department of Medical Microbiology & Parasitology, Faculty of Medicine & Health Science, Universiti Putra Malaysia, Serdang, Malaysia

**Keywords:** tocotrienols, Alzheimer's disease, Parkinson's disease, therapeutic potential, neuroprotective, reverse nerve damage, antioxidant, mitochondrial shield

## Abstract

Neurodegenerative disorders, such as Parkinson's and Alzheimer's disease, are claimed to be of major concern causing a significant disease burden worldwide. Oxidative stress, mitochondrial dysfunction and nerve damage are the main reasons for the emergence of these diseases. The formation of reactive oxygen species (ROS) is the common chemical molecule that is formed from all these three interdependent mechanisms which is highly reactive toward the neuronal cells. For these reasons, the administration of tocotrienols (T3s), which is a potent antioxidant, is proven to cater to this problem, through *in vitro* and *in vivo* investigations. Interestingly, their therapeutic potentials are not only limited to antioxidant property but also to being able to reverse the neuronal damage and act as a shield for mitochondria dysfunction. Thereby, T3s prevents the damage to the neurons. In regards to this statement, in this review, we focused on summarizing and discussing the potential therapeutic role of T3s on Alzheimer's and Parkinson's diseases, and their protective mechanisms based on evidence from the *in vitro* and *in vivo* studies. However, there is no clinical trial conducted to prove the efficacy of T3s for Alzheimer's and Parkinson's subjects. As such, the therapeutic role of T3s for these neurodegenerative disorders is still under debate.

## Introduction

Over the past 25 years, neurodegenerative disorders, such as Alzheimer's (AD), Parkinson's (PD), and Huntington (HD) diseases, are on the upsurge due to longer life expectancy, as age is one of the risk factors when contracting any one of these diseases (1). Due to the complex pathogenesis of neurodegenerative disorders, detecting and treating the disorders remain as a challenging task despite the existence of a wide range of modern technologies and medicine (2). With this in mind, there is a continuous interest among researchers to overcome such problems by using natural compounds as an alternative and complementary medicine (3). Besides consumed in their natural forms, these compounds can be modified to enhance or suppress certain properties such as stability, efficacy and bioavailability (4). Many of them serve as lead compounds for designing new drugs with desirable pharmacological activity.

The usage of natural compounds, such as tocotrienols (T3s) (3), curcumin (5), flavonoid apigenin (6), crocin (7), rosmarinic acid (8), green tea catechins (9), resveratrol (10), and hesperidin (11), has been reported to be effective against several types of neurodegenerative disorders such as AD (3, 5–11), PD (3, 5–11), diabetic neuropathy (6, 11), stroke (6), epilepsy (7), cerebral ischemia (10), HD (10, 11), and multiple sclerosis (11). These compounds are capable of reducing the beta amyloid (Aβ) aggregation by increasing monoamine secretion in AD and PD (8), preventing abnormal accumulation of Aβ and α-synuclein in AD and PD (9), acting as an antioxidant against AD (3, 5–11), improving blood flow to the cerebral, and improving cognition and performance in AD, PD, HD, and multiple sclerosis (11). Interestingly, being a naturally existing substance, they have been reported to exhibit minimal adverse effects (3). Recent epidemiological data indicates that persons with high plasma levels or a high dietary intake of the combination of all natural vitamin E forms had a lower incidence of dementia or AD. In these, tocotrienols are more effective than tocopherol in neutralizing certain free radicals, given the fact that all vitamin E congeners show antioxidant activity (12, 13). Currently, more than 1/3 of the top selling pharmaceutical medicines are natural compound origin and labeled as traditional medicine. However, there are not many clinical trials that have proven the efficacy of these compounds. Yet, day by day, there are more and more natural product-based formulations being introduced to the market (14).

### Neurodegenerative Disorders

The nervous system is made up of neurons comprising of nerves and specialized cells that transmit signals between various parts of the body. The transmission of signals greatly influences the normal coordination of body activity. A small alteration in the signal transmission may cause neurological changes that lead to neurodegenerative disorders. AD and PD are two of such diseases that have distinct pathologies yet share some similar features in pathogenesis. There are some common cascades of neuronal events such as protein toxicity and oxidative stress prior to progressive neurodegeneration in these patients. Toxicity of alpha-synuclein in PD and Aβ42 or tau proteins in AD are some of the common neuronal alternations detected among those patients (15). In terms of cell signaling, both show overlapping events in the activation of oxidative stress, glycogen synthase kinase-3 beta, mitogen-activated protein kinases (MAPK), and cell cycle re-entry (16).

In addition, the development of both the PD and AD occurs in the brain. Many factors, such as aging, exposure to environmental toxin and unhealthy lifestyle, attribute to the development of these diseases (17). Both of the diseases are linked with mitochondria dysfunction in association with increased oxidative stress. Exponential oxidative stress, together with DNA mutation, leads to mitochondria dysfunction which is much implicated in the aging process (18). In AD, oxidative stress leads to the activation of amyloidogenesis pathway which elevates the development and deposition of amyloid peptide (19). Equally in PD, oxidative stress leads to misfolding of α-synuclein protein that causes neurodegeneration; loss of dopaminergic neurons and loss of dopamine in the brain (20).

### Alzheimer's Disease

AD is a form of dementia characterized by deposition of neurofibrillary tangles and amyloid plaques in the brain which resulted in loss of neurons and synapses (21). AD can also be defined by the deterioration in everyday activity due to progressive waning in two or more cognitive functions and verified by anomaly in clinical and neuropsychological testing (22). AD can be categorized into 4 stages; (1) preclinical stage of AD, (2) Mild Cognitive impairment due to AD and (3) moderate AD and (4) Severe AD (23). The pre-clinical stage of AD is characterized by slight memory loss, alterations in the cortex and hippocampus, but no significant hindrance in everyday life: the mild cognitive impairment due to AD displays early symptoms of AD such as memory loss, disorientation and mood swings with amyloid accumulation in the brain, moderate AD shows increase memory loss, failure to recognize friends and family members, with amyloid accumulation and loss of neurological function, and finally, the severe AD stage leads to complete failure recognizing in friend and family, loss of cognitive activities as the cortex area is fully affected (24). Collectively, the progression of AD can be monitored by determining the accretion neurochemical such as amyloid, tau proteins and neurofibrillary tangles (25).

Approximately 60–80% of dementia cases are due to AD (26). According to the world Alzheimer's report, it is expected that more than 130 million people tend to suffer from this condition by the year 2050 (27). Those who are diagnosed with AD tend to live up to 8 years. In the United States, AD has been recognized as the 6th leading cause of death (28). Increasing age is known to be a major risk factor for AD patients above 65 years old, but the exact cause of AD remains unknown. Current available treatment is said to be very limited and failed in reversing the progression of the disease, although some may improve the symptoms temporarily. AD development is closely related to two distinct mechanisms in the nervous system, namely deposition of extracellular Aβ and accumulation of intracellular tau protein in the brain (29).

Amyloid beta (Aβ) is part of a larger protein, the transmembrane protein, amyloid precursor protein (APP). Aβ peptide is comprised of up to 43 amino acids in length as a result of amyloid precursor protein (APP) cleavage via β- and γ-secretase. In normal condition, the monomers and oligomers of Aβ are degraded by protease and cleared in the brain (30). Defective clearance of Aβ from aberrant APP cleavage results in the accumulation of Aβ at the extraneural tissues. When this happens, the soluble Aβ monomers polymerize initially into soluble oligomers, and then into larger insoluble fragments such as Aβ42, which precipitated as amyloid fibrils (31). Elevated level of amyloid fibrils in extracellular brain region will suppress excitatory synaptic activity at the postsynaptic level, particularly those in the form of oligomers will impair the synapse transmission. This happens because oligomers will bind to the pre- and post-synaptic neurons. Thereafter, loss of long term potentiation, synapse damages, and neuronal apoptosis will occur (32).

Tau protein is found in the neurons and its primary role is to ensure the stabilization of the internal microtubules. Tau protein accumulation happens due to the degeneration of neurofibrils. Initially, tau is aggregated as a paired helical filament, twisted around each other, forming paired helical filaments. These deposits interfere with cellular functions by displacing organelles. By altering the spacing of microtubules, axonal transport is impaired, thereby affecting the nutrition of dendrites and axon terminals. This will start at the entorhinal cortex, preceding to the hippocampus, and then to the connection cortex at later time (33). These will result in microtubules instability, mitochondrial damage, and pathogenic signaling that alter the molecular motor phosphorylation. Eventually, the associated motor functions are altered (34).

### Parkinson's Disease

PD is a progressive neurodegenerative disorder that affects movements. It usually results in trembling, imbalance, and stiffness (35). Clinically, features such as rest tremor, bradykinesia, postural instability and akinesia are most well-identified symptoms of PD (36). Other non-motor symptoms include psychiatric impediments such as control disorders, anxiety, depression, dementia and sleep disorders (37). The clinical symptoms are prominent during the third stage of the disease where the death of dopaminergic neurons in the substantia nigra pars compacta and noradregenic neurons at the locus coeruleus occurs (38). This neurological abnormality is pathologically characterized by the accumulation of α-synuclein protein in the form of Lewy bodies and Lewy neurites (39). Meanwhile, in association with PD, the neurochemical metabolites, such as dopamine, 5-hydroxytryptamine (5-HT), gamma-aminobutyric acid (GABA), and glutamate, have been found to be lower in PD patients (40). These neurotransmitters can be used to track the development of PD.

Epidemiological data indicate that men are 50% more prone to get this disease in comparison to women (35). Although age is considered to be the primary risk factor for PD, <10% of patients develop the disease before the age of 50. In certain cases, the etiology of this disease is closely related to certain gene mutations which include synuclein, alpha (SNCA), Parkin RBR E3 ubiquitin protein ligase (PARK2), Parkinson's disease protein 7 precursor (PARK7), PTEN-induced putative kinase 1 (PINK1), ubiquitin carboxy-terminal hydrolase L1 (PARK5), and leucine-rich repeat kinase 2 (LRRK2) (41). There are a few known mechanisms that are related to the development of PD.

One of the mechanisms is due to the imbalance of ROS level which arises from the metabolism of dopamine. In the presence of iron, dopamine breakdown may occur spontaneously. Dopamine is an unstable compound which can form quinones and hydrogen peroxide (H_2_O_2_) by auto-oxidizing. H_2_0_2_ may form more active hydroxyl radical (?OH) by reacting with irons or oxygen (O_2_). Dopamine quinones can react with cysteine in sulfhydryl groups, especially reduced glutathione (GSH), a ROS scavenger, resulting in decreased GSH and increased ROS levels (42). On the other hand, monoamine oxidase (MAO) catalyzes the breakdown of dopamine in a reaction that generates H_2_O_2_. The H_2_O_2_level is considered as safe to cells until cytotoxic OH are produced excessively through Fenton reaction. ROS can lead to structural and functional alterations in proteins, DNA and lipids. Lipid damage causes a loss of membrane integrity while increasing permeability to ions such as calcium in substantia nigra that will trigger excitotoxicity (43).

Another mechanism closely linked to PD is the loss of substantia nigra dopaminergic neurons associated with the involvement of intraneuronal inclusions known as Lewy bodies. The Lewy body is an unusual aggregation or clumping of the α-synuclein protein (44). In the brain, native α-synuclein is often deployed without a specified tertiary structure, although it can be present in stable tetramers that resist aggregation in aqueous solutions. Usually, α-synuclein folds through its N-terminal into α-helical structures upon interaction with negatively charged lipids, such as the phospholipids that make up cell membranes. In PD, α-synuclein adopts an amyloid-like structure rich in β-sheet that is vulnerable to aggregation. Several mechanisms, including serine 129 phosphorylation, ubiquitination, and C-terminal truncation, have been speculated for the conformational changes that lead to abnormal α-synuclein aggregation. α-synuclein can interact with the mitochondrial membrane and accumulate within the organelles. This contributes to the damage of complex I activity, eventually to mitochondrial dysfunction and increased oxidative stress. This is because the interaction between oligomeric α-synuclein and the translocase of outer membrane 20 (TOM20) mitochondrial receptor may lead to impairment of the machinery importing mitochondrial protein leading to decreased respiration which results in excessive formation of ROS (45).

## Vitamin E

**Figure 1:**
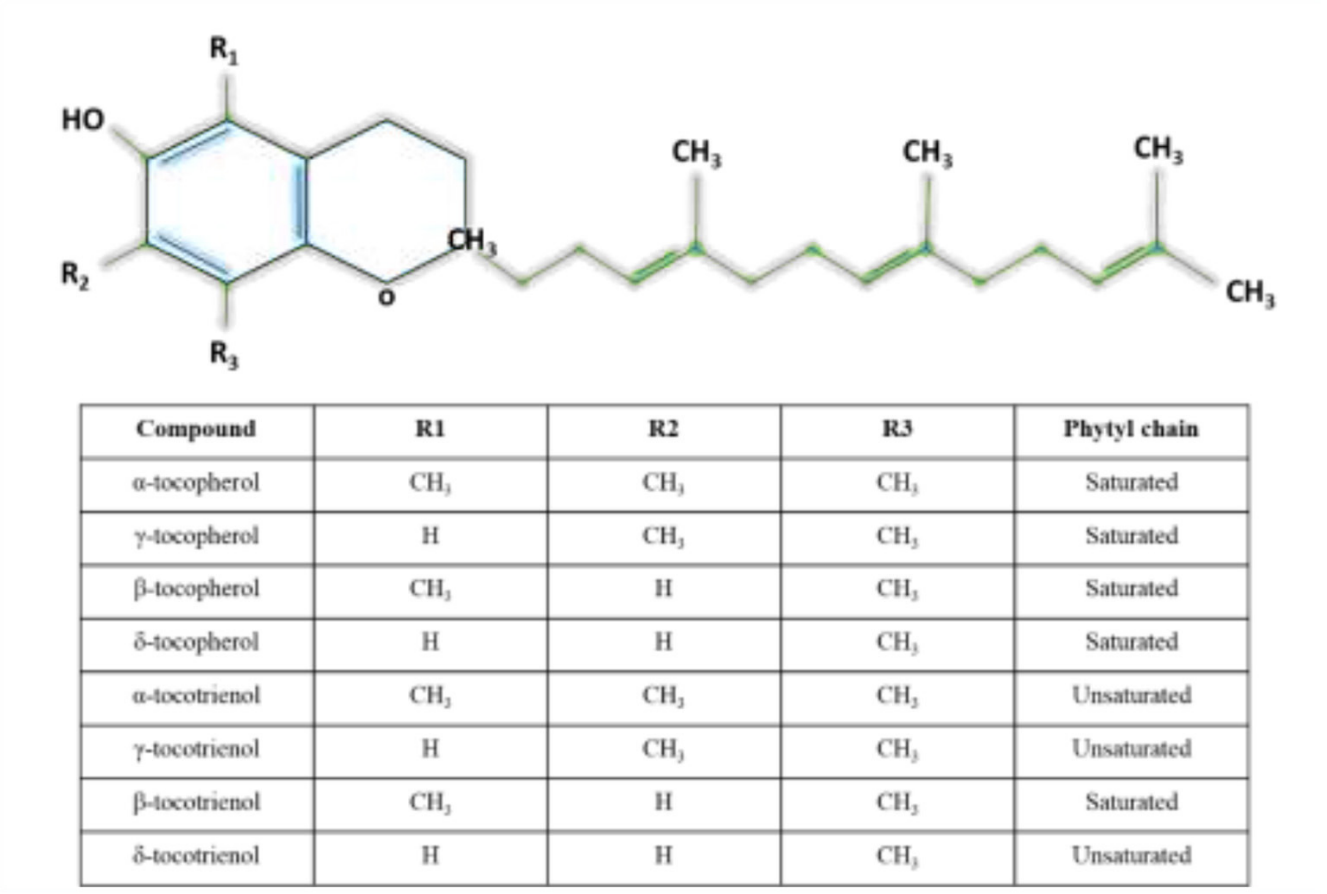
Structures of tocotrienols and tocopherols.

Vitamin E is a lipid soluble compound that consists of eight members which are known for their antioxidant property. The members are divided into tocopherols and T3s such that each group is further divided into α, β, γ, and δ forms (46). Structurally, T3s and tocopherols share a chromanol head with an aliphatic side chain. Tocopherols have a phytyl tail (saturated) side chain, while T3s have an isoprenoid (unsaturated with three double bonds) side chain (47). The differences among α, β, γ, and δ forms are the position and number of the methyl groups on the chromanol head. Figure 1 shows the different forms of T3s and tocopherols. Vitamin E can be found in nuts, vegetable oils and seeds, while palm oils, rice grain and annatto seed are a rich in T3s (47).

### Biological Activities of Tocotrienols

The functional properties of tocopherols and T3s can be differed from one another: T3s have been reported to exhibit more potent activities than tocopherols. Apart from possessing neuroprotective effects, T3s have cholesterol-lowering capacity (48) and anti-tumor property (49). Despite the lower bioavailability, antioxidant activity of T3s outranges tocopherols. Previous study has shown that α-T3 has greater peroxyl radical scavenging capability in the liposomal membrane in comparison to the α-tocopherol due to the presence of unsaturated side chain which enhances the even distribution of T3 in the bilayer membrane. The even distribution facilitates the interaction between chromanol ring of α-T3 with lipid radicals (50). T3s also move much more rapidly than α-tocopherol within lipid vesicles (51).

## Therapeutic Mechanisms of Tocotrienols

AD and PD arise from distinct pathogenesis. Yet, they share some common molecular damages such as oxidative stress, mitochondrial dysfunction and neurodegeneration. In consideration to this, T3s have been shown to be effective in combating these damages. Tables 1, 2 summarizes the effect of T3s in AD and PD in regards to *in vitro* and *in vivo* trials.

**Table 1:** Effects of T3s on AD in *in vitro* and *in vivo* studies.

**References**	**Intervention**	**Gender and age**	**Model and strain**	**Findings**
Yatin et al. (62)	T3&Tocopherol, dose not specified	18-day-old; gender not specified	Sprague Dawley rat's fetus; embryonic hippocampal neuronal cells of Aβ peptide-treated rat	• Reduced ornithine decarboxylase activity in the neuronal cells. • Decreased spermidine uptake by neuronal cells. • Increased polyamine metabolism in the neuronal cells.
Sung et al. (92)	T3&Tocopherol (member unspecified), 2 IU/g/d × 6 months, Regular chow	5 and 14 months; gender not specified	Tg2576 mice	• Reduced lipid peroxidation in hippocampus. • Reduced Aβ levels and amyloid deposition in hippocampus. • Reduced brain oxidative stress in hippocampus.
Conte et al. (93)	T3&Tocopherol, 2 IU/g/d × 8 weeks, Regular chow	11 months; female	Tg2576 mice	• Reduced lipid peroxidation in brain. • Reduced Aβ levels and amyloid deposition in brain. • Improved spatial learning ability. • Decreased ROS in brain.
Yao et al. (94)	T3&Tocopherol, 10 mg/ℓ/d × 7 months, Drinking water	8 months; female and male	Tg2576 mice	• Decreased Aβ1-40 and Aβ1-42 in hippocampus. • Suppressed brain inflammation and ROS in brain hippocampus. • Reduced isoprostane 8,12-iso-iPF2α (iPF_2α_-V) in brain hippocampus.
Grimm et al. (66)	10 μM α-T3, 24 h	Not applicable	SH-SY5Y wild type cells; APP695 transfected SH-SY5Y cells	• Reduced Aβ degradation in N2a cells. • Reduced ROS level. • Reduced cholesterol and cholesterol esters. • Reduced γ-secretase activity in neuro 2a (N2a) cells.
Damanhuri et al. (54)	TRF, 200 mg/kg/d × 6 months, Oral gavage	9 months; male	Wild type and double transgenic mouse B6C3-Tg (APPswe, PS1dE9) 85Dbo/Mmjax with C57BL/6J	• Increased superoxide dismutases (SOD) activity in hippocampus. • Increased catalase (CAT) activity in hippocampus. • Decreased DNA damage in the brain. • Increased in antioxidant effect.
Ibrahim et al. (95)	TRF, 1%, 0.01%, 0.001% v/v × 24 h	Not applicable	Cell free assay; Human neuroblastoma cell line SH-SY5Y	• Disrupted Aβ42 aggregation. • Reduced Aβ42 fibrils formation. • Inhibited Aβ oligomerization.
	TRF, 60 mg/kg/d × 15 months, Oral administration	5 months; female and male	APPswe/PS1dE9 double transgenic mice (APP/PS1)	• Absence of cytotoxic effect. • Reduced Aβ deposition in the hippocampus. • Reduced thioflavin-s-positive fibrillar type plaques in hippocampus and cortex.
Durani et al. (61)	TRF, 5 mL/kg/d × 15 months, Oral administration	5 months; female and male	APP/PS1 double transgenic mice	• Increased exploratory activity. • Enhanced spatial learning, memory and recognition memory. • Increased level of neurotransmitters such as (acetylcholine, L-tyrosine, L-glutamic acid, and L-aspartic acid) metabolism in medial pre-frontal cortex, striatum and hippocampus.
Mohamed et al. (57)	T3 (members unspecified), 100 mg/kg/d × 8 weeks, Oral administration	Male; age not specified	White albino rats (Sprague Dawley); 2-vessel occlusion rats	• Increased neurons with compact cellular structure of stratum pyramidale. • Increased well-demarcated cell membrane, clear cytoplasm, and distinct nucleus in brain. • Decreased Isoprostane F_2_ (Iso-F_2_) in brain.
Hamezah et al. (55)	TRF, 60 mg/kg/d × 10 months, Oral gavage	5 months; male and female	APPswe/PS1dE9 double transgenic (Tg) mice (AβPP/PS1)	• Inhibited ERK and c-Src in MAPK pathway. • Increased glial fibrillary acidic protein (GFAP) in hippocampus. • Decreased Interleukin enhancer-binding protein 2 (ILF-2), ACTR10 protein, APP and PTPRA protein expression in hippocampus.
Wan Nasri et al. (84)	TRF, 200 mg/kg/d × 6 months, Oral gavage	9 months; male	Double transgenic male mouse B6C3-Tg (APP swe, PS1dE9) 85Dbo/Mmjax with C57BL/6J	• Elevated gene expression of Ataxia telangiectasia mutated (ATM), and Calcium Voltage-Gated Channel Subunit Alpha1 B (CACNA1B). • Downregulated messenger RNA (mRNA) processing, focal adhesion-PI3K-Akt-mTOR signaling, epidermal growth factor receptor 1 (EGFR1) signaling, p53 signaling, T cell receptor signaling, Tumor necrosis factor-alpha (TNF-α) NF-kB signaling, and MAPK signaling.

**Table 2:** Effects T3s on Parkinson's disease in *in vitro* and *in vivo* studies.

**References**	**Intervention**	**Gender and age**	**Model and strain**	**Findings**
Hagl et al. (96)	T3, tocopherol and γ-oryzanol, 150 mg/kg/d × 30 days, Oral gavage	Not applicable	PC12 cells; SNP-treated brain cells of guinea pigs fed	• Increased mitochondrial respiration in brain. • Increased dynamin-related protein 1 (Drp1) and fission 1 (fis1) proteins. • Increased mitochondrial mass.
Nakaso et al. (97)	10–1,000 nM γT3 and δT3 48 h	Not applicable	MPP^+^-treated SH-SY5Y cells	• Cytoprotective against MPP+. • Activation of PI3K/Akt signaling pathway in cell line. • Formation of caveola. • Stimulation of translocation of estrogen receptor beta (ERβ) from cytosolic to the perinuclear space.
Hagl et al. (69)	T3, tocopherol and γ-oryzanol, 340 mg/kg/d × 3 weeks, Oral gavage	Female NMRI mice; 18 months	sodium nitroprusside (SNP)-treated mice	• Protected brain cells against nitrosative stress. • Increased mitochondrial content. • Increased mitochondrial membrane potential. • Increased adenosine triphosphate (ATP) production.
Nakaso et al. (98)	α-T3 and δ-T3, 100 μg/kg/d × 6 days, Oral gavage	Female and male; age not specified	MPTP induced C57BL/6 mice	• Increased expression of estrogen receptor-α in olfactory bulb and cerebellum. • Reduced estrogen receptor-β expression in the brain. • Preservation of neuron cells from oxidative damage. • Recovery of spontaneous activity.
Matsura (63)	γ-T3 and δ-T3, 48 h	Not applicable	MPP+-treated SH-SY5Y cells	• Cytoprotective against 1-methyl-4-phenylpyridinium (MPP+). • Activated MAPK, PI3K/Akt. • Translocation of Akt from cyctosolic to plasma membrane.
	γ-T3 and δ-T3, 6 days	Not specified	MPTP mouse PD model	• Recovered voluntary performance. • Reduced oxidative stress. • δ-T3 protection against MPTP-induced neurotoxicity.
Kumari (67)	α-T3 and γ-T3, 200 mg/kg/d × 28 days, Oral gavage	Male SD rats; 14 weeks old	6-OHDA-treated rats	• Delayed disease progression. • Diminished motor deficits. • Restored Parkinson's disease 6 (PARK 6) gene. • Restored gene expression of tyrosine hydroxylase (TH), Dopa-decarboxylase (DDC), Solute Carrier Family 18 Member A2 (SLC18A2), solute carrier family 6 member 3 (SLC6A3) and nuclear receptor related 1 (NURR1) genes. • Neuroprotective against dopaminergic neurons.

### Tocotrienols as an Antioxidant

**Figure 2:**
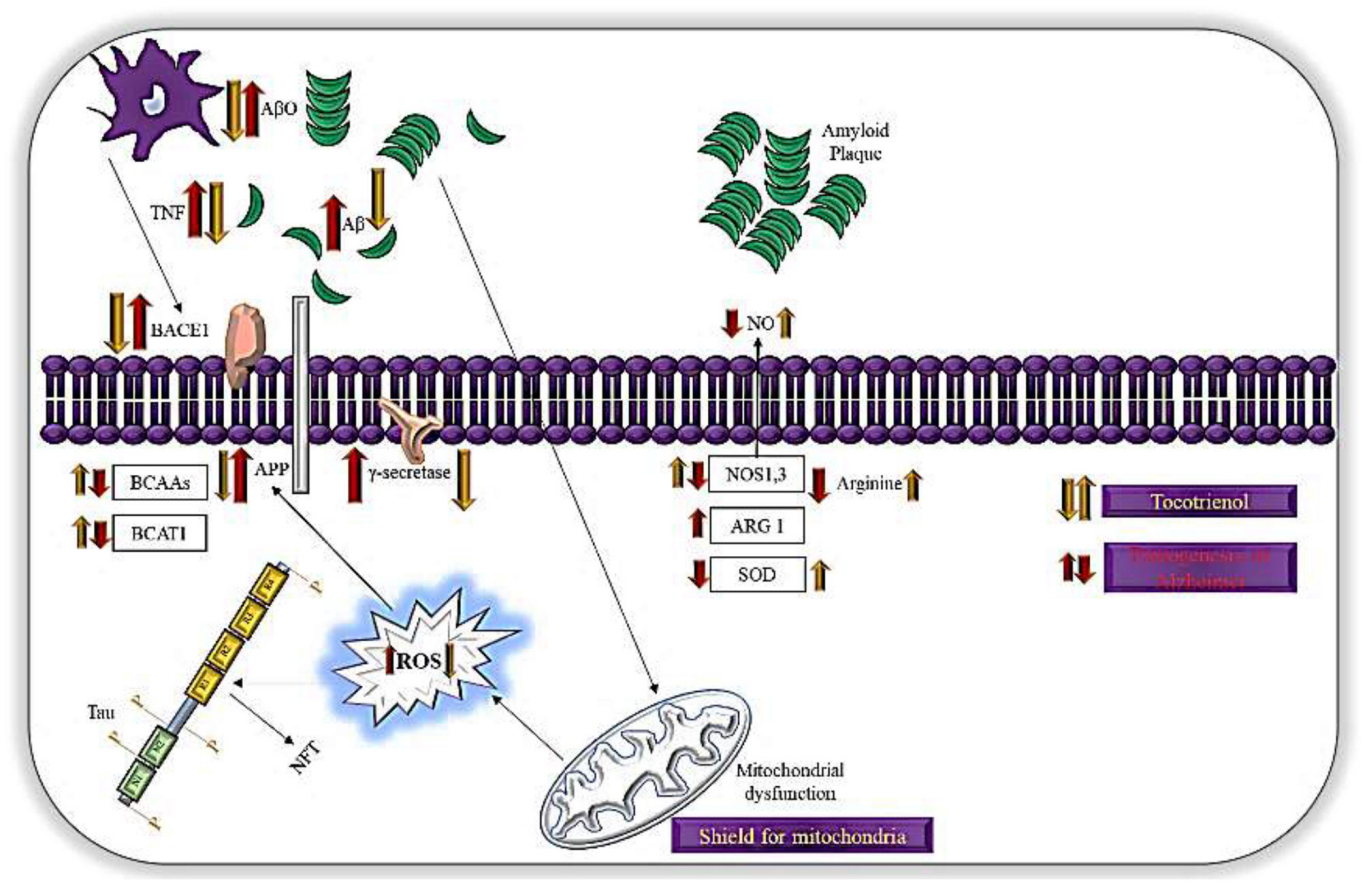
Mechanisms of action of T3s in AD. T3possess antioxidant activities, which mitigate oxidative damage in neurons. T3 shown to increase the translocation of BACE1 to the cellular membrane leading to enhanced non-amyloidogenic APP processing and impaired γ-secretase dependent APP cleavage. This mechanism also due to lipid raft disruption. Mitochondria is a main generator for ROS while T3 reduces lipid peroxidation and act as shield for mitochondria, preventing formation of ROS.

The brain requires high level of oxygen for its optimal function. Due to this, it is the primary organ susceptible to oxidative insults by free radicals (45). The formation of Aβ hinders the activity of antioxidant enzymes, specifically glutathione peroxidase (GPx), superoxide dismutase (SOD), and catalase (CAT) (52). Aβ binds to CAT, SOD and GPx with high affinity and inhibits H_2_O_2_ breakdown by these enzymes (52). Eventually, this will result in elevation of oxidative stress. As a consequence, proteins, lipids, and DNA will start to oxidize. Normally, SOD functions to dissociate excess superoxide anion into hydrogen peroxide (H_2_O_2_) and O_2_, while glutathione peroxidase (GPx) and CAT will then reduce the H_2_O_2_ into water. In the absence of these enzymes, H_2_O_2_ will be transformed into hydroxide (^−^OH) (53). In relation to this, α-T3 has been proven to increase the SOD activity in AD mice's brain (54). This clearly defines the reason behind the disruption of Aβ42 aggregation and reduction in Aβ deposition at the hippocampus of APPswe/PS1 AD mice (37, 39). This has been illustrated in Figure 2 which refers to the mechanisms of action of T3s in AD. Similarly, Hamezah et al. (55) noticed a decrease of APP in the hippocampus of APPswe/PS1 mice treated with T3s-rich fraction (TRF). In addition, TRF hinders the formation of Aβ fibrils and oligomers in a cell-free assay, and decreases the accumulation of Aβ in the hippocampus and pre-frontal cortex of AβPP/PS1 mice (55).

Khor et al. (56) have shown that TRF has the capacity to upregulate gene expressions of GPx and CAT in senescent myoblasts. Furthermore, TRF decreases the activity of lipid peroxidase and inhibits its binding toward the DNA in neuronal cells of myoblasts, preventing oxidative damage to the DNA (56). T3s have the capacity to hinder the production of other reactive species such as nitric oxide molecule (3), particularly peroxynitrite (56) and nitrosamines (3). This mechanism is further supported by Mohamed et al. (57) who noticed a reduction in isoprostane F_2_ by T3, a by-product of lipid peroxidation, in hippocampal cells prepared from 2-vessel occlusion neurodegenerative model rats. Damanhuri et al. (54) have shown that TRF increases CAT enzyme activity but has no effect on GPx activity in the hippocampus of AβPP/PS1 mice.

The existence of unsaturated side chain in γ-T3 eases the penetration of γ-T3 into tissues (58). This further ensures it is evenly distributed in the surface of the lipid layers of the cell membrane. With the aid of lipoprotein lipases or lipoprotein endocytosis mediated by receptors, tissue absorbs γ-T3 more effectively. Thus, peroxyl radicals will be removed rapidly as the even distribution most likely enhances the interaction of chromanols with lipid radicals (58). TRF is evidenced in protecting cytochrome P450 from oxidative damage (59). A study done by Tan et al. (60) proves that the location of α-T3, which is closer to the surface of the membrane, enhances the recycling process of chromanols. This serves as the main reason for continuous reduction of oxidative stress.

Most researchers agreed that T3s act as an antioxidant in AD mice (29, 31, 35–39). The protective mechanisms of T3s against AD has led to the reduction of Aβ, ROS, oxidative stress marker (Iso-F_2_), or lipid peroxidation marker (iPF_2a_-VI). In fact, the ability of T3s to act as an antioxidant is the primary reason to reverse certain age-associated symptoms such as memory. Durani et al. (61) noticed a drastic increase in spatial, exploratory, and recognition memory in AβPP/PS1 AD mice treated with TRF.

Yatin et al. (62) observed reduction in ornithine decarboxylase and spermidine by T3 in Aβ-treated embryonic hippocampal neuronal cultures of rat. The findings suggest that T3 acts as a free oxidative radical scavenger that enhances polyamine metabolism. The need for cellular polyamine biosynthesis and the ability to take up extracellular polyamines are increased when there is an increase demand for polyamines. A regulatory protein, an antizyme, is regulated by polyamine uptake and its biosynthetic rate. Antizyme synthesis is the cell's natural way to defend itself by slowing down transport of ornithine decarboxylase activity against over-accumulation of polyamines to toxic levels (62). This is another mechanism of T3 as an antioxidant agent.

**Figure 3:**
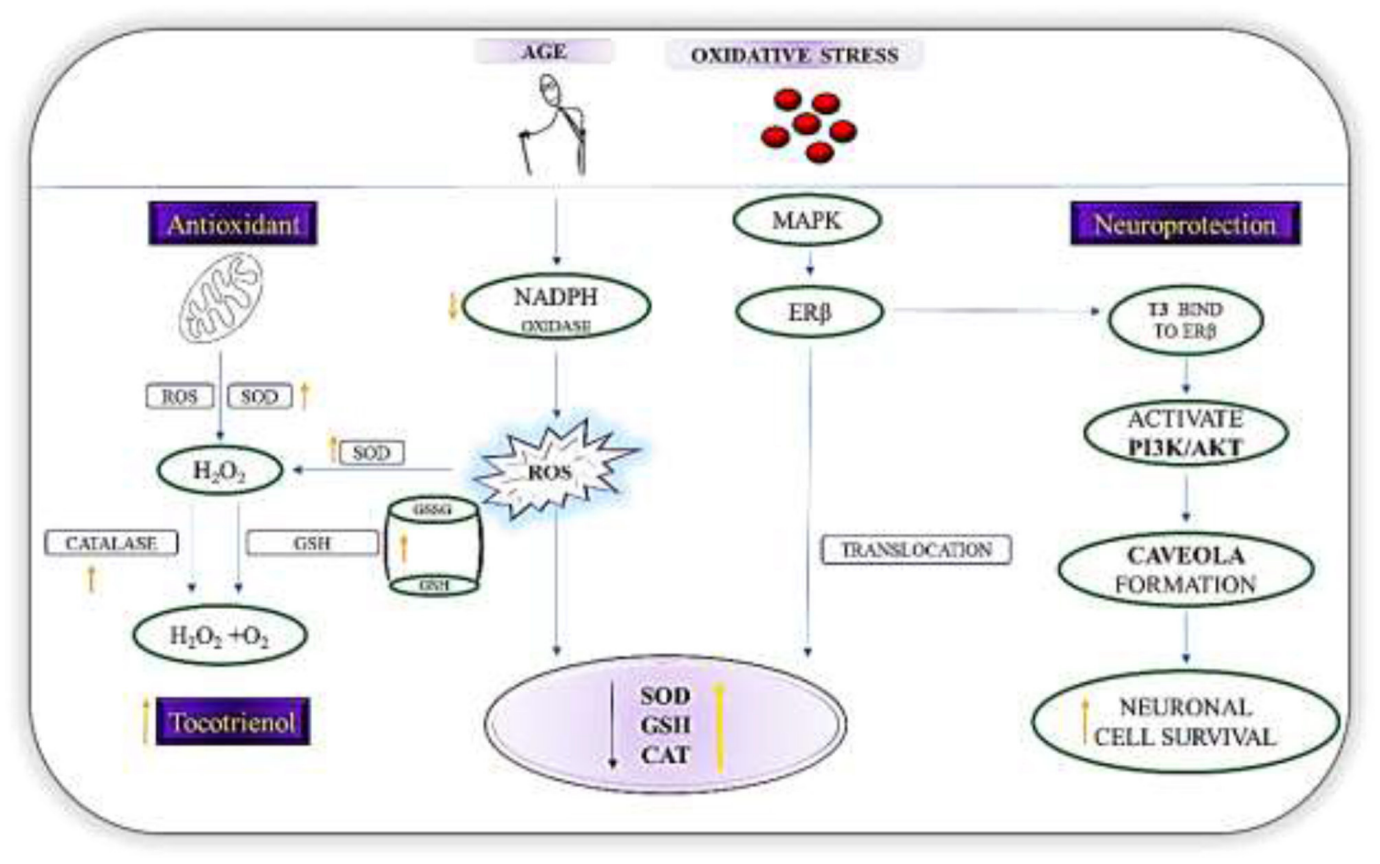
Mechanisms of action of T3s in PD. Diagram shows the role of T3 as a first line defense antioxidants in PD by increasing the level of SOD, GSH, and catalase via the nuclear translocation of ERβ and breakdown of H_2_O_2_ to harmless molecules (H_2_O_2_/alcohol and O_2_). T3, via the regulation of NADPH oxidase reduces reactive oxygen species formation. In addition, T3s binds directly ERβ, which then triggers the signal transduction pathway and activates PI3K/Akt pathway. Resultantly, caveola will be formed. These initial events constitute an estrogen dependent non-genomic pathway that is neuroprotective.

Matsura (63) notices reduction of oxidative stress in 1-methyl-4-phenylpyridinium ion (MPP^+^)-treated SH-SY5Y cells when the cells are supplemented with γ- and δ-T3 within 48 h. Comitato et al. (64) report that T3 is effective against PD-related toxicities such as MPP^+^ through the binding of γ- and δ-T3 to ERβ which will lead to a marked activation of PI3K/Akt signaling pathway. Activation of PI3K/Akt signaling reduces ROS by negatively regulating expression of the downstream proteins such as Forkhead Box Protein O1 (FOXO1) and Caspase 3 (65). Comparably, Nakaso et al. (66) observe the activation of PI3K/Akt signaling via ERβ binding by γ- and δ-T3 in MPP^+^ treated SH-SY5Y cells. The similar mechanism is believed to be underlaid in the study done by Kumari (67, 68) who notice a prevention in the loss of dopamine neurons in α- and γ-T3 treated groups. Figure 3 summarizes the action of T3 in PD as an antioxidant agent.

Hagl et al. (69) observe the T3's ability to protect brain cells against sodium nitroprusside (SNP)-induced nitrosative stress in aged NMRI mice. Nitrosative stress is a common pathology found in PD patients. It facilitates S-nitrosylation of neuroprotective proteins and compromises their function, thereby leading to cognitive impairments (70). This is because SNP is a potent neurotoxicant as it generates nitric oxide (NO), cyanides, and free irons. This could stimulate typical programmed cell death changes such as formation of the apoptotic nuclei, activation of caspase-3/7 and−9, decrease of the Bcl-2/Bax ratio, decrease level of matrix metalloproteinases (MMP), and release of cytochrome c in neuronal cells. In dopaminergic cells, this may trigger cytotoxicity that leads to dopaminergic cell death caused by SNP (71). At the same time, NO may combine with superoxide to form peroxynitrite, which rapidly causes protein nitration or nitrosylation, lipid peroxidation, DNA damage and neuronal cell death (72). In this scenario, γ- and δ-T3 reduce nitrosative stress by minimizing the activity of nitric oxide synthase and then leading to the reduced formation of nitric oxide (73). This is achieved by downregulating TNF-α, TNF-1β, NF-κB and caspase-3 activities (72).

Furthermore, in AD, an increase in 3-hydroxy-3-methylglutaryl coenzyme A (HMG CoA) reductase is a common pathology. Along with tocotrienol consumption, there are inverse associations that have been witnessed between the incidence of AD and blood levels of the HMG CoA reductase. T3s has the ability to inhibit the processing and nuclear localization of SREB-2, the transcriptional factor for HMG CoA reductase and farnesyl pyrophosphate (FPP) synthase, and further promotes HMG CoA reductase degradation. As a result, tocotrienols decrease the pool of FPP and geranylgeranyl pyrophosphate (GGPP), potentially slowing the progression of prenylation-dependent AD. T3s' anti-inflammatory properties contribute to their protection against Alzheimer's disease. In sum, T3s inhibit the processing and maturation of SREBP-2 and increase the breakdown of HMG CoA reductase, making them a preventive agent against AD (74).

### Tocotrienols as a Cell Signaling Mediator

Neurotransmission of excitatory glutamatergic neurons is essential for the survival of neurons and synaptic plasticity. Normally, this neurotransmission mechanism takes place with N-methyl-d-aspartate receptor (NMDAR). However, small alteration in this activity, mainly elevation of NMDAR, could result in excitotoxicity of glutamatergic neurons, thereby leading to neuronal cell apoptosis in AD (75). In PD, slight increase in glutamate concentration due to loss of nigral dopaminergic neurons triggers the glutamate overactivity (76). In both disorders, the increased level of glutamate eventually damages the neurons, especially the HT4 neural cell (77). Aside from this, in consequence of the glutamate rise, extracellular signal regulated kinase (ERK) (78) and 12-lipoxygenase (LOX) (79) will be activated. Hence, there will be a sudden rise in the level of calcium ion (Ca^2+^), depletion of GSH, dysfunction of mitochondria and eventually neuronal death (79). T3s, especially α-T3, are found to provide neuroprotection in glutamate-induced death of neuron cells at nanomolar concentrations beyond its antioxidant nature (58).

Cellular antioxidant defense is compromised by a GSH-depleted state, accompanied by increased susceptibility of the cell to ROS. T3s, through an antioxidant-independent mechanism, are able to inhibit the activation of pp60^c−src^ kinase. This happens as nanomolar level of α-T3 suppresses glutamate-induced early activation of c-Src kinase (58). Src family kinases are a group of genes that are involved in the regulating of cell growth. The Src family kinase product, c-Src, is strongly expressed in the brain. Overexpression of c-Src may stimulate the activation of glutamate leading to neurodegeneration caused by glutamate (80).

Apart from this, T3s enhance the re-entrance of injured cells into S phase (DNA synthesis) and G2/M phase (DNA repair and recovery) of cell cycle (81). This is to reverse the glutamate-induced apoptosis of the neuronal cell (58). Inversely, since brain contains a very high level of arachidonic acid (AA), a major component of polyunsaturated fatty acids (PUFAs), it is highly susceptible to oxidative metabolism. T3s have acted as mediators for AA, altering metabolism of AA (58). In this, α-T3 will interact directly with the enzyme that suppress AA anabolism (82). This happens as T3s are able to cleave the membrane phospholipid bilayer via cytosolic phospholipase A_2_ (58). The carboxyl (COOH) terminal at the AA site will enter the 12-LOX solvent cavity while the catalytic site is accessed. It is therefore possible that the binding position of α-T3 prevents access of the AA substrate to the active 12-LOX site. As so, LOX-12 is inhibited. Thus, 12-LOX inhibition protects Aβ-induced toxicity in the corticol neurons (82). The reduction in receptor-type tyrosine-protein phosphatase alpha (PTPRA) protein expression by TRF, as shown by Hamezah et al. (55), further confirm the role of TRFs as a neuroprotective agent (55) via the inhibition of ERK and c-Src in MAPK pathway in APPswe/PS1 mice. PTPRA is perceived as a crucial mediator for synaptic plasticity and neuronal migration and association with memory. At the same time, PTPRA plays a central role as an activator of Src family kinases (83). In regards to this case, TRFs have been proven to be effective in decreasing the excess level of PTPRA at the hippocampus of AβPP/PS1 mice, thereby exerting neuroprotective effect (55).

On the contrary, Wan Nasri et al. (84) observe a significant increase of *ATM* and *CACNA1B* expression in AβPP/PS1 mice treated with TRF. One of the primary functions of ATM is to provide sufficient duration for DNA repair in the cell cycle. This is a post mitotic process which prevents replicative stress on the neurons (85). Expression of ATM signaling is feasible for replication process of neurons, thereby reducing neuronal apoptosis. *CACNA1B* encodes the presynaptic neuronal voltage-gated calcium channel Cav2.2/N-type pore-forming subunit. As so, this *CACNA1B* is essential for neurotransmission (86). In other words, the expression of *CACNA1B* enhances excitatory transmitters (87). From here, we can speculate that in the AD mice, the action potential generated by excitatory neurotransmitters has been re-established upon being treated with T3s.

### Tocotrienols as a Shield for Mitochondria Dysfunction

Mitochondrial dysfunction is an early hallmark of AD and PD. Extracellular or intracellular Aβs are imported into the inner membrane of mitochondria in the neuronal cell of AD brain. The progressive accumulation of mitochondrial Aβ leads to impaired energy metabolism, functional defects in key respiratory enzymes, increased mitochondrial ROS and altered mitochondrial biogenesis. This Aβ will bind to proteins in the mitochondria, such as cyclophilin D (CypD) and amyloid-binding alcohol dehydrogenase (ABAD), leading to mitochondrial dysfunction which will contribute to neuronal damage and cognitive impairment (88). Meanwhile, mutations in the mitochondrial DNA (mtDNA) are common feature of PD. The accumulation of mtDNA mutations will increase the relative levels of somatic mutations. mtDNA mutations lead to bioenergetic mitochondrial deficiency in the neurons of substantia nigra. With the accumulation of mtDNA mutations and increase in oxidative stress, reduction in mitochondrial function decreases cellular bioenergetics and favors α-synuclein aggregation, leading to the development of Parkinsonism (89).

T3s have been speculated as effective in shielding mitochondria and microsomes from oxidative stress. Mitochondria has been reported as the major target of oxidative stress due to its major role as an energy powerhouse which generates free radicals as by-products (90). Cytochrome c is generated excessively when ROS is produced inside the mitochondria (91). Cytochrome c will then come into contact with caspase-9 and Apaf-1. Thus, caspases-associated apoptosis is stimulated. Caspase-3 activation results in DNA-fragmentation and apoptosis (90).

T3s function not only limited to decreasing the activity of lipid peroxidation, but also act as a protective shield against mitochondrial dysfunction. γ-T3 exhibits positive effects such as improved mitochondrial function on aged mice through the elevation of ATP level and membrane potential. This is confirmed through the increase expression of mitochondrial transcription factor A which serves as an initiator of mtDNA duplication and further leads to increased mitochondrial function (81). Another study reveals significant reduction in mitochondrial levels of pro-apoptotic proteins such as Bid, Bax, and Bad upon being exposed to T3s in the hippocampus of AD mice (99). Concurrently, Park et al. (100) observe subsequent increase in mitochondrial levels of anti-apoptotic proteins such as Bcl-2 and Bcl-xL when α-T3s is used as a treatment in the glutamate-treated primary hippocampal neurons. Studies done by Hagl et al. (62, 92) have shown that T3s act as mitochondrial shield by increasing mitochondrial mass, content, membrane potential and respiration in the brain of APPswe/PS1 mice and APP695-treated SH-SY5Y cells. Therefore, it can be speculated that the stabilization and integrity of the mitochondrial membrane can be strengthened when a small amount of T3s is being used as treatment.

## Tocotrienols-Based Supplements in the Market

The beneficial effects of T3s on neurodegenerative disorders have been proven through *in vitro* and *in vivo* studies; and no adverse effect or dose toxicity of T3s has been reported in these studies. The positive outcomes have urged the pharmaceutical industries to formulate T3s into tablets, powder, or syrups for consumption as health supplement. Nevertheless, the efficacy of these products on human health remains to be tested. Most of these products are labeled as antioxidants, while others claimed to improve neurological function and prevent cognitive aging. With the evidence from cell and animal studies, more and new T3s-based products are expected to be released by the pharmaceutical companies, but clinical trials will be needed to convince the consumers in purchasing these products. Table 3 shows the currently marketed T3 products which claimed to be neuroregenerative.

**Table 3:** T3s-based products for neuroregeneration.

**Formulation**	**Brand and company**	**Claim**	**Dosage**	**References**
δ-T3	DeltaGold®, American River Nutrition	Prevent age-related cognitive decline	3.5 mg	(101)
α,β,δ, and γ-T3	Naturale, DavosLife	Cell wellness and protection	50 mg	(102)
Natural α,β,δ, and γ-T3	EVNol^TM^, ExcelVite	Antioxidant in various system	30–50 mg/day	(103)
Bioenhanced natural α,β,δ, and γ-T3	EVNol Suprabio^TM^, ExcelVite	Antioxidant in various system	30–50 mg/day	(103)
Water dispersible natural α,β,δ, and γ-T3	EVNolMax^TM^, ExcelVite	Antioxidant in various system	30–50 mg/day	(103)
Water dispersible natural α,β,δ, and γ-T3	EVNolBeV^TM^, ExcelVite	Antioxidant in various system	30–50 mg/day	(103)
Natural α,β,δ, and γ-T3	Tocobeads®, ExcelVite	Antioxidant in various system	30–50 mg/day	(103)
γ-T3	Tocotrol™ L50P, Fuji chemical industry	Improve neurological function	50–100 mg	(104)
30% Palm T3 Powder	TocoTab®, Fuji chemical industry	Improve neurological function	50–100 mg	(104)

The bioavailability of T3 is the definition which incredibly impacts the degree of assimilation. The same group of analysts also demonstrate that self-emulsifying frameworks, that produce better beads of emulsion, noticeably upgrade the retention of T3 by ~2–3-folds higher compared to non-emulsified definition. It is also observed that the slack time required for the emission of bile salts to emulsify the T3 in arrange to encourage their retention are moderately shorter with the nearness of self-emulsifying frameworks (13).

## Safety, Limitation, and Future Prospective of Tocotrienols

The low bioavailability of T3s, particularly in the form of oral formulations, is one of the greatest challenges in the therapeutic field (101). Due to this, the recommended dose to consume is 50–100 mg of T3s in a day to experience the beneficial effect (103). In terms of cost, extraction of T3s can be very expensive. In relation to this issue, approximately more than US$8000 is needed to extract 1 g of γ-T3 from natural sources (46). This is the main reason why most of the study is limited to tocopherols or synthetic tocopherols, instead of focusing on T3s. Additionally, it is really hard to synthesize T3s due to the existence of three double bonds in the isoprenoid side chain (101). So far, there is no reported dose limiting toxicity that has been recorded for T3s consumption. However, it is advisable for those with blood disorders to avoid consuming T3s as they possess anticoagulant effect (81). There are limitations including the forms of vitamin E that are not clarified in some areas. As such, the composition of vitamin E supplement might not reflex the genuine composition within the diet. At present, there is no reliable evidence of adequacy of vitamin E within the avoidance or treatment of individuals with AD; in this way, more investigation is required. However, the preclinical evidence supporting the use of antioxidants to prevent or slow AD is strong. There is clear evidence for increased oxidative damage in the brain of AD patients and numerous potential sources of excess free radicals that may contribute to this damage. To add on, the market for T3s remains robust. By the year 2026, it is expected that the market value for T3s might reach up to USD522.0 million (105). In considering this, continuity in research, especially in bioavailability, is highly recommended to fully utilize T3s for the prevention of neurodegenerative disorders in the future.

## Conclusions

*In vitro* and *in vivo* studies have confirmed the neuroprotective potential of T3s against neurodegenerative diseases such as AD and PD through the actions as antioxidant, shield of mitochondria damage, and neuroprotectant against neuronal cell death. These effects are expected to be validated by clinical trials. The market value for T3s as supplement for preventing neurodegenerative diseases is high, while approaches that lower their extraction cost and enhances bioavailability are anticipated as sensible focus of future research in T3s.

## Author Contributions

RN: conceptualization, software, data curation, and visualization. RN and NS: methodology and writing review and editing. HB and NS: validation and investigation. RN and HB: formal analysis and writing original draft preparation. PK: resources. HB: supervision and funding acquisition. NS: project administration. All authors have read and agreed to the published version of the manuscript.

## Funding

This study was funded through the grant provided by Fundamental Research Grant (FRGS/1/2020/SKK0/UPM/02/4) in the form of database subscription. The funders do not have any contribution and decision to publish or preparation of the manuscript.

## Conflict of Interest

The authors declare that the research was conducted in the absence of any commercial or financial relationships that could be construed as a potential conflict of interest.

## Publisher's Note

All claims expressed in this article are solely those of the authors and do not necessarily represent those of their affiliated organizations, or those of the publisher, the editors and the reviewers. Any product that may be evaluated in this article, or claim that may be made by its manufacturer, is not guaranteed or endorsed by the publisher.
